# American Medical Society in Paris

**Published:** 1855-03

**Authors:** 


AMERICAN MEDICAL SOCIETY IN PARIS.—Dr E. W. Johnston, a graduate of the University, has been recently elected president of this Society for the third time. He writes us that the number of American physicians in Paris is much smaller than usual. Dr. Johnston has promised us a monthly resumé of medical matters in Paris, which will be no doubt highly acceptable to our readers.

At the sitting of the Paris Academy of Sciences, the committee on the very numerous communications respecting the Asiatic Cholera, reported that not one of them deserved to be taken into serious consideration. Since 1849, a prize of a hundred thousand francs has been offered in vain, for a prescription which would cure the disease in the immense majority of cases. It will now be given for a positive, certain indication of the causes of the Asiatic Cholera, so that, by the removal of them, it should disappear; or for the discovery of a prophylactic (a sure preventive), such as vaccination is for the small pox. There is, likewise, a prize of five thousand francs for a demonstration of the existence, in the terrestrial atmosphere, of any matter or animalcule operative in the production or propagation of epidemic diseases.

The Academy of Medicine held its annual sitting a few days ago; a copious report was submitted on the subjects, and the merits of the memoirs to which the prizes for the year 1854 have been awarded. At this sitting the stated eulogy embraced three celebrated men. It may be noted that nearly all the great French physicians and surgeons of this century were of the humblest parentage, and passed through severe struggles; and, notwithstanding prodigious professional successes, few of them died rich.

*Antidote to Prussic Acid.*—A remarkable case of recovery after taking a large dose of Prussic acid is reported in the English journals. The victim immediately swallowed half an ounce of aromatic spirit of ammonia, with a little water, and then had administered to him some solution of crystals of sulphate of iron, trusting to the ammonia previously swallowed for the formation of an insoluble compound of the acid with the oxides of iron. Cold douche was also applied, more sulphate of iron and spirits of ammonia were administered, and in a short time perfect recovery took place.

*Electricity.*—During a violent storm which lately burst over Paris, the electric fluid entered a room in which was seated a man who had long been suffering from paralysis, which deprived him of the power of speech. It set fire to the bed-curtains and did other damage in the room; but instead of injuring the infirm man it restored him to his speech and health.—*Academy of Sciences, French Institute.*

